# Revealing Nucleic Acid Mutations Using Förster Resonance Energy Transfer-Based Probes

**DOI:** 10.3390/s16081173

**Published:** 2016-07-27

**Authors:** Nina P. L. Junager, Jacob Kongsted, Kira Astakhova

**Affiliations:** Department of Physics, Chemistry and Pharmacy, University of Southern Denmark, Campusvej 55, 5230 Odense M, Denmark; nijun12@student.sdu.dk (N.P.L.J.); kongsted@sdu.dk (J.K.)

**Keywords:** nucleic acid, mutation, fluorescence, FRET, molecular beacon, binary probe, computational strategies, in vitro hybridization, J0101

## Abstract

Nucleic acid mutations are of tremendous importance in modern clinical work, biotechnology and in fundamental studies of nucleic acids. Therefore, rapid, cost-effective and reliable detection of mutations is an object of extensive research. Today, Förster resonance energy transfer (FRET) probes are among the most often used tools for the detection of nucleic acids and in particular, for the detection of mutations. However, multiple parameters must be taken into account in order to create efficient FRET probes that are sensitive to nucleic acid mutations. In this review; we focus on the design principles for such probes and available computational methods that allow for their rational design. Applications of advanced, rationally designed FRET probes range from new insights into cellular heterogeneity to gaining new knowledge of nucleic acid structures directly in living cells.

## 1. Introduction

Nucleic acids are essential for all known forms of life. In the form of deoxyribonucleic acid (DNA) and ribonucleic acid (RNA), they function in encoding, transmitting and expressing genetic information [1]. To ensure these functions, nucleic acids actively interact with each other and other biomolecules, change conformations and move within living cells. So far, obtaining data on nucleic acids in a biologically relevant context has been hindered by the lack of ultra-sensitive methods and reliable probes [2]. The recent progress in biophysics and chemistry of nucleic acids has provided an opportunity to open up the black box of nucleic acid structure and interactions directly in cells and to quantify nucleic acids in diverse biological environments [2].

Upon hybridization, two complementary nucleic strands bind to each other, forming a duplex. Currently, nucleic acid hybridization is seen as a thermodynamically driven process which is promoted by hydration forces and stacking interactions between nucleobases [3]. A practical outcome of this framework is design of synthetic DNA and RNA probes to target nucleic acids in vitro or in vivo. Moreover, nucleic acid modifications are finding growing applications in the preparation of target-specific probes [4]. This theoretical framework is confirmed by extensive experimental data in vitro, at concentrations above 50 nM and for unmodified and modified sequences [5]. A forthcoming challenge for the theory and design of oligonucleotide probes is that natural DNA and RNA are long biopolymers which are present in complex intracellular matrices at extremely low concentrations, often only a couple of molecules per cell [1,2].

Establishing design strategies is crucial for oligonucleotide probes that will target and sense nucleic acids and, in particular, single-nucleotide polymorphisms (SNPs) or, in other words, mutations. So far, diverse parameters of modified oligonucleotides have been addressed separately. This includes binding affinity assessments by thermodynamic models [6], SNP detection using modified analogues [7] and reports on the influence of dyes on the solubility and kinetics of hybridization [8]. However, these data are not integrated, are limited to very few synthetic nucleotide analogues and have a rather weak theoretical background. Moreover, current calculations of oligonucleotide properties do not predict fluorescence response, mismatch sensitivity or the influence of a probe’s concentration and microenvironment on target binding and signaling [9]. Therefore, a trial and error approach is still often applied for design of fluorescent probes. As a result, expensive and time-consuming synthetic procedures and detection may be of no use, due to the potential of sensitivity and specificity of the probes. Simultaneously, demand for effective fluorescent probes is rapidly growing [10].

**Figure 1:**
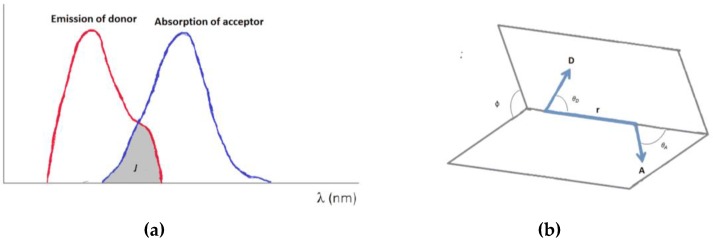
Main parameters of FRET: (**a**) Spectral overlap, J, of donor emission and acceptor absorption (necessary for FRET); (**b**) Transition dipole orientation of the donor D and the acceptor A.

Fluorescence is a convenient method of nucleic acid detection [11]. Among other optical effects applied in nucleic acid studies, Förster resonance energy transfer (FRET) is a quantum phenomenon occurring between two dye molecules, a donor and an acceptor, which are in close proximity (Figure 1). The excitation energy absorbed by the donor molecule is transferred non-radiatively to an acceptor fluorophore through long-range dipole-dipole interactions through space (Figure 1b). Although the theory of long-range molecular interactions was introduced by Theodor Förster in 1948 [11,12], the first biological applications of this phenomenon only started in the 1970s, applied primarily for protein research [12]. For nucleic acid studies, oligonucleotide synthesis by the phosphoramidite method developed in the 1980s stimulated the use of FRET probes for biotechnology and genomics later in the 1990s [13,14].

In this review we describe recent advances in the design of effective FRET probes. Herein we mainly focus on probes that detect clinically and biologically actionable nucleic acid mutations. Many FRET probes and assays described in the literature potentially can be expanded to the detection of SNP although this is not described in original papers. Herein we introduce such examples as well. An important and novel aspect of this review is the description of recent advances of molecular modeling and quantum chemical approaches for design of effective FRET probes. As demonstrated below, synergetic application of advanced computational chemistry with the chemistry of modified oligonucleotides and biophysics can provide breakthrough scientific and diagnostic settings addressing challenging biological and biomedical tasks.

## 2. Förster Resonance Energy Transfer-Based Probes in Nucleic Acid Research and Molecular Diagnostics

### 2.1. Theory of FRET

The theory of FRET has been described in detail in a plethora of publications and textbooks and is not a main focus of this review. The brief description given below has the purpose of providing the reader with a short introduction to the theory of FRET as a tool for detection of nucleic acid variants in diverse environments. The interaction between the donor and acceptor in FRET is understood as a non-radiative transfer between molecules through dipole-dipole interactions. First, the donor is excited from the ground state (GS) to an excited state (EX). Then, the acceptor receives the excitation energy and relaxes back to the GS with or without fluorescence [15]. For well-separated molecules the efficiency (*E*) of FRET depends on two parameters, the distance, *r*, between the acceptor and the donor, and the Förster distance, R_0_. This can be expressed as follows [16]:

(1) $$E=\frac{R_{0}{}^{6}}{R_{0}{}^{6}+r^{6}}$$

However, in many cases the efficiency in the actual situation is more complex. The Förster distance is defined as the distance at which there is 50% FRET efficiency. Most FRET probes have a Förster distance within 10–100 Å [15] and the expression for the Förster distance (in Å) is given by:

(2) $$R_{0}=0.211{\left[\frac{\kappa^{2}\phi_{D}J}{n^{4}}\right]}^{1/6}$$

where n is the refractive index of the medium, *φ_D_* is the quantum yield of the donor fluorophore and *J* is the spectral overlap as detailed later. κ^2^ describes the orientation factor of the coupled donor and acceptor transition dipole moments, relative to each other:

(3) $$\kappa^{2}={\left(\sin\theta_{D}\sin\theta_{A}\cos\phi -2\cos\theta_{D}\cos\theta_{A}\right)}^{2}$$

The orientation parameter, κ^2^, assumes values in the range from 0 to 4 [17], where 0 corresponds to a perpendicular orientation between the donor and acceptor transition dipoles, 1 is parallel and 4 a collinear orientation, and as such the dipole orientation plays an important role in improving FRET performance. However, κ^2^ is often approximated to be 2/3 which is the value obtained for an isotopically orientation of the coupled transition dipole moments. Importantly, some fluorophore pairs differ from that approximation, which has to be taken into account in the fluorophore design [18].

The spectral overlap can be described further as:

(4) $$J=\int F_{D}\left(\lambda \right)\varepsilon_{A}(\lambda )\lambda^{4}d\lambda$$

where *F_D_* is the normalized emission energy spectrum and *ε_A_* is the molar absorption coefficient of the acceptor; λ is the wavelength. There are two forms of FRET donor-acceptor pairs giving rise to either hetero-FRET or homo-FRET. Hetero-FRET is the most commonly used; here the donor and acceptor fluorophores differ whereas in homo-FRET they are identical. An overlap between the emission wavelength of the donor and the excitation wavelength of the acceptor is required for resonant energy transfer. In homo-FRET the stoke shift (the difference between the band maxima of excitation and emission of a dye) needs to be small to create an efficient FRET whereas for hetero-FRET the stoke shift need to be relatively large to ensure an excitation of only the donor and not the acceptor.

Requirements for the occurrence of FRET within nucleic acids can be extrapolated from the Förster equations (Equations (1) and (2)). First, a spectral overlap between the donor emission and acceptor absorption has to be present (Figure 1a). Second, the donor needs to have a sufficient molar extinction coefficient within the nucleic acid environment and third the distance and orientation between the donor and acceptor must be sufficient for Forster energy transfer. Distances below approximately 10 Å might lead to quenching or activation of other mechanisms such as e.g., Dexter transfer [11].

### 2.2. Challenges and Appeal of FRET Probes in Modern Nucleic Acid Research

Attachment of FRET pairs to DNA and RNA probes is an attractive strategy for measuring hybridization of two strands in vitro, in cell lines and in vivo. However, multiple functionalities of nucleic acids bring complications into the aforementioned FRET theory. First, interactions between the nucleotide bases and the fluorophore could be responsible for a quenching effect which is not caused by FRET. For example, quenching by nucleobases is observed when ssDNA labeled with a single fluorescein is annealed to an unmodified complementary strand [19,20,21]. Second, fluorophores are generally attached through relatively short and rigid linkers and cannot be assessed as freely rotating transition dipoles but should be considered as non-isotropically orientated. This might have a critical effect on FRET-pair with a relatively short separation (10–20 Å) [16,18]. Also, a rise in temperature can have a negative effect on the energy transfer efficiency [22], whereas incorporation of the third dye acting as a relay station between the two other FRET dyes improves the transfer [23].

Importantly, parts of the fluorophore-labeled DNA probe can participate in collisional and static fluorescence quenching. These non-FRET-based mechanisms can mimic the fluorescence-quenching effects of FRET [24,25]. On the other hand, the fluorescence suppression caused by collisional (dynamic) and static (complex formation) quenching [24,25] is often used in its own right in DNA probes labeled with various dark quenchers that do not satisfy the essential spectral overlap criteria for FRET [26,27]. In both cases, unlike FRET, the direct contact of the fluorophore and the quencher is required [24]. Consequently, both dynamic and static quenching occur at distances shorter than those optimal for FRET; thus, both static and dynamic quenchers may be considered contact quenchers [24].

In DNA probes with nonlinear configurations that constantly change their shape, the situation could include all of the abovementioned. For example, in “breathing” (constantly opening and closing) hairpin constructs that have the donor and acceptor positioned on the opposing free DNA ends, both dyes could go through a wide range of distances, making it possible for all mechanisms of quenching to participate in the fluorescence suppression [19].

Interactions of FRET probes with nucleic acids become even more complicated when the detection is to be performed directly in cells. Unlike short oligonucleotides, intracellular DNA and RNA targets are long biopolymers which are present in complex intracellular matrices at extremely low concentrations, often only a couple of molecules per cell [1,2]. For the last three decades, low abundance of nucleic acids has been approached using enzymatic amplification including polymerase chain reaction (PCR) [28]. PCR and similar techniques utilize enzymes which, in a templated fashion, create high amounts of shorter, replicated nucleic acids starting from just a few molecules. PCR and other amplification methods have resulted in tremendous scientific and technological advances, although over the past decade there has been a growing concern regarding adequacy of the data provided by enzymatic amplification. First, it has been discovered that not every nucleic acid sequence can be amplified [28]. Moreover, stoichiometry of the initial sample is affected by enzymatic reactions, and the reaction cannot be carried out in living cells [29,30,31]. Finally, polymerases make mistakes upon synthesis of nucleic acids [32,33]. These are crucial obstacles for detection of SNP by amplification [32].

Very recently, creative imaging methods have approached biomolecules in the original biological context, reaching single-molecule resolution [34]. In imaging with optical methods, a target biomolecule can be reproduced visually in its original form. Among other detection techniques for imaging, fluorescence, and FRET in particular, is a convenient, highly informative method [35]. In several works, folding of nucleic acids has been studied by two-photon microscopy [36,37]. A special variant of multiphoton fluorescence microscopy, the two-photon method uses red-shifted excitation light to excite fluorescent dyes and simultaneously to reduce the background.

Among other super-resolution imaging methods, stimulated emission depletion (STED) methods have the advantage of the highest resolution and compatibility with diverse fluorophores, given that they have high fluorescence quantum yields and photostability [38]. In a recent example, STED-based nanoscopy has been proposed for studies of DNA-protein interactions by FRET at a resolution less than 50 nm [39]. STED nanoscopy creates super-resolution images by the selective deactivation of fluorophores, minimizing the area of illumination at the focal point, and thus enhancing the achievable resolution for a given system.

Besides challenging detection, bringing FRET probes into live cells dramatically complicates interpretation of results [40,41,42,43,44]. Especially when a single resolution limit of detection is approached, advanced computational methods and controls are applied to see the probe’s localization. Here, emission wavelength and probe photostability become crucial [40,41,42,43,44]. In many assays, it is warranted that the former does not overlap with cells’ autofluorescence and other labels applied to distinguish cellular compartments. The latter allows performance of imaging in living cells by wavelength-separation measurements.

In general, the Förster parameters do not take environmental effects such as pH change into account. Since the donor determines the maximal energy available for transfer, a good quantum yield and brightness [16] is required. Furthermore, bleaching of the donor decreases the quantum yield and, hence, the limit of target detection.

Finally, in cells, it is not sufficient to have high affinity and specificity for a target nucleic acid sequence [45,46,47,48]. The probes must also be able to penetrate barriers, such as membranes, to reach the target [49]. They must also be stable under physiological conditions, resistant towards degradation, have minimal cytotoxicity, minimal off-target effects and minimal ability to distort cellular functions, as well as being simple, easy to detect with fluorescence and having a high signal to background ratio [45,49].

### 2.3. Design and Application of FRET Probes for Nucleic Acid Research

#### 2.3.1. Design

**Figure 2:**
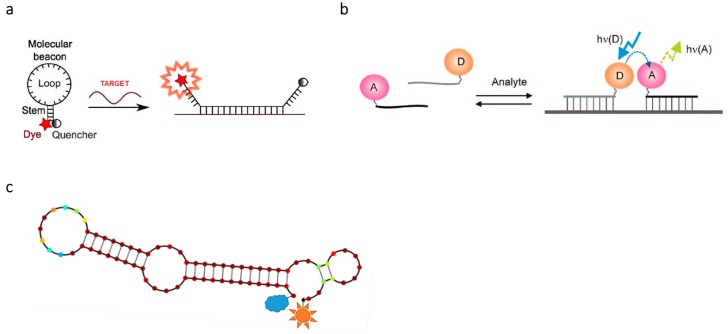
Design of FRET probes for nucleic acid detection: molecular beacons (**a**); binary probes (**b**); RNA aptamer labelled with fluorophore and quencher (shown as a star and cloud, respectively) (**c**).

Figure 2 shows the general design approach for FRET probes that have recently been successfully applied in the detection of nucleic acids. These FRET probes can be divided into two classes: unimolecular and bimolecular. The unimolecular class involves the use of one probe that undergoes conformation change to generate FRET, while the bimolecular class requires two probes that interact [19].

Molecular beacons (MBs) are single-stranded unimolecular oligonucleotides, typically 20–30 nucleotides long (Figure 2a). They were introduced by Tyagi and Kramer in 1996 [50]. In the absence of a target the terminal ends are held together as a stable stem (5–7 nt). The rest of the oligonucleotide possesses a hairpin loop which is antisense, i.e., complementary, to the target. A fluorophore is typically attached to the 5′ end and a non-fluorescent dye, called a quencher, is at the 3′ of the probe. Non-fluorescent quenching dyes are also called dark quenchers. Like any other FRET acceptor, the absorption spectrum of the dark quencher has to have an overlap with the donor emission spectrum, but unlike a fluorescent acceptor, the transferred energy is quenched by the dark/non-emissive acceptor [51]. In the presence of the target the MB will undergo conformational change: the stem will open, the antisense sequence will hybridize to the target and the distance between the fluorophore and the quencher will increase significantly. The length of the stem affects its selectivity. Probes with longer stems require that the hybrid formed with the target should be quite energetically favorable, which makes it more selective for a perfectly matched target and thereby ideal for SNP genotyping [52]. One disadvantage of MBs is the appearance of false positive results due to, among other factors, nonspecific opening of the stem, incomplete quenching and cleavage of backbone which leads to release of the dye [53].

An example of bimolecular probes is binary probes, also called adjacent probes, a pair of two linear antisense probes (Figure 2b). One probe has a donor fluorophore attached to its 5′ end, and the other probe has an acceptor fluorophore attached at or near the 3′ end. Under hybridization of the two probes, they come into close proximity to each other, which allows FRET to occur [54]. In the absence of a target, no FRET is observed because the distance between the fluorophores is too big [55]. The optimal Förster distance between the two fluorophores depends on their spectral properties. For example, the FRET pair pyrene-perylene has a Förster distance of 20–30 Å in nucleic acid probing, compared to the regularly used fluorescein-rhodamine pair with a Förster distance above 40 Å. Using both pairs, the probes have been successfully used in vivo and in vitro, although with a relatively low signal-to-noise ratio for the fluorescein-rhodamine pair [56].

Another type of FRET probe is aptamers. Aptamers are RNA or DNA molecules that, through a well-defined secondary structure, have the ability to bind to a target with a high affinity and specificity (Figure 2c). Targets of aptamers are very diverse and include small molecules, proteins (e.g., secreted factors, intracellular proteins and membrane receptors), peptides or nucleic acids [57,58]. Aptamers can be created in a SELEX procedure, where probes get developed through repeating rounds. Starting with a pool of random sequences, each round of SELEX increases the aptamer’s affinity and specificity for the target. FRET has found extensive use in the field of DNA and RNA aptamers, i.e., in the study of protein-protein interactions [59] and protein-nucleic acid interactions under SELEX, along with testing of obtained aptamers [60,61].

**Figure 3:**
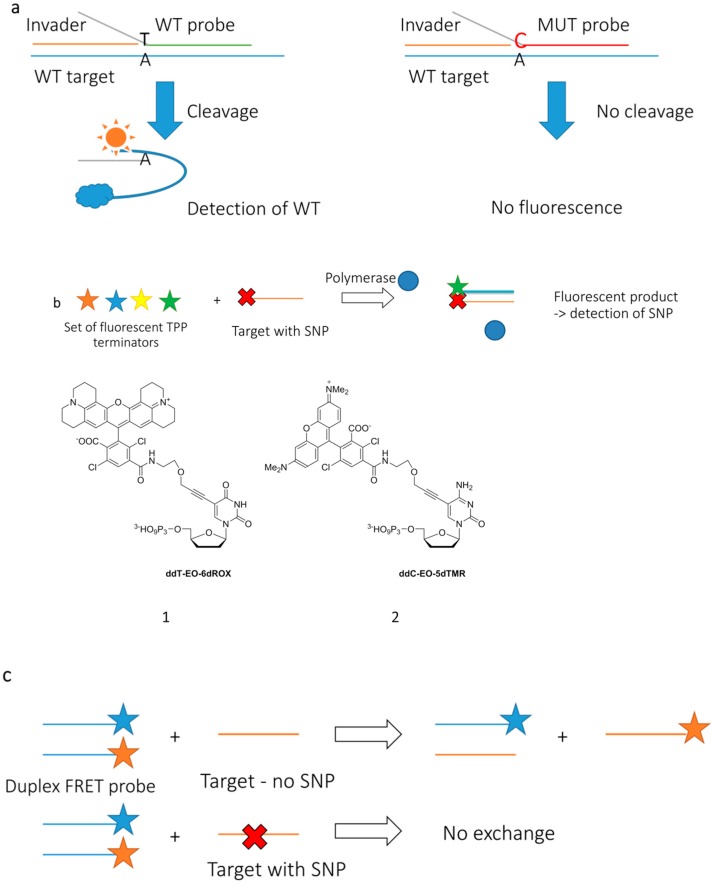
Mismatch sensitive FRET probe designs [61,62,63,64,65]: Invader probes (**a**); fluorescently labelled triphosphate terminators (**b**) and duplex probes (**c**). WT = wild type; MUT = mutant.

Specific designs for oligonucleotide FRET probes developed for the detection of mutations can employ either the disruption (Invader probes, Figure 3a) or creation (template-directed dye-terminator incorporation, or TDI assay, Figure 3b) of energy transfer systems [62,63]. The former interact with nucleic acid duplexes based on their high affinity, whereas the latter utilize enzymatic nucleic acid synthesis for the detection of a target. A particular example of nucleotide terminators are compounds **1**–**2** (Figure 3b). Because of lacking 3′-OH group, upon incorporation into the growing DNA chain these compounds block further sequence elongation. Resulting products are detected using fluorescence signal of the dyes that are attached to corresponding terminator, i.e., rhodamines attached to position 5 of thymine and cytosine in **1** and **2**, respectively [63]. Another approach uses a double-stranded FRET probe formed by two complementary oligonucleotides (Figure 3c) [64,65,66]. One of the oligonucleotides is 5′ end-labeled with the donor fluorophore, and the other has a 3′-attached acceptor. The rationale of the assay is to detect the target sequence by competitive hybridization of one of the oligonucleotides forming the probe duplex. When the initial probe duplex is disrupted, because of the hybridization of one of the oligonucleotides to the target sequence, the quenching of the donor is stopped and its fluorescence becomes observable. In a demonstration of the practical utility of the assay, fluorescein was used as a donor and either pyrenebutyrate or sulforhodamine 101 was used as an acceptor [65]. The assay detected as little as 10 pM of the target DNA sequences. The use of FRET probes of similar design allowed for the detection of hybridization events occurring in live cells [66].

#### 2.3.2. Approaching Challenges of SNP Detection by Modern FRET Probes: Chemistry of Backbone Modifications and Advanced Fluorescent Dyes

**Figure 4:**
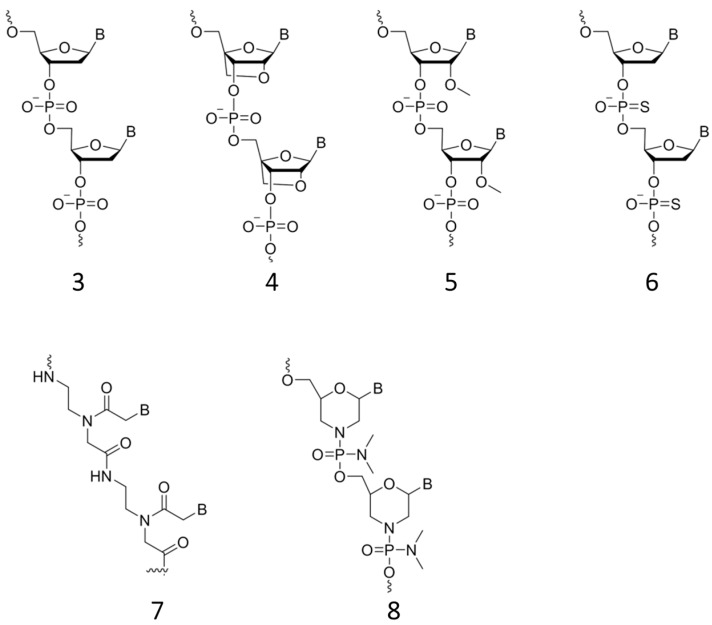
Chemical structures of synthetic oligonucleotides containing modified backbones.

Modifications of oligonucleotide backbones can improve stability of FRET probes to enzymatic degradation and dramatically improve sensitivity to SNP. A modification that improves affinity and specificity towards target DNA and especially RNA is locked nucleic acid (LNA) (Figure 4, structure **4**). LNA is a nucleic acid analogue with a methylene linkage between the 4′-C and 2′-*O* positions. The binding structure ring holds the structure and the ambient nucleic acid into a rigid structure. LNA was discovered in 1998 by the groups of Wengel [45] and Imanishi [46], independently. Another approach is the use of 2′-*O*-methylribonucleotides (2′-*O*-Me-RNA) (Figure 4, structure **5**). Similarly to LNA, when bound to RNA they exhibit a higher binding affinity, enhance the hybridization rate and are able to discriminate between matched and mismatched RNA targets [67]. 2′-*O*-Me-RNA probes also show nuclease resistance [68]. One disadvantage of 2′-*O*-Me is the inability to penetrate cells and hence the requirement of injection into living cells [69].

Phosphorothioates (PT) are a modification of the backbone in which a non-bridging phosphate oxygen is replaced with a sulfur (Figure 4, structure **6**). Incorporation of PT into MBs leads to more stable products, less likely opening and nonspecific fluorescence. According to a recent report, such probes only slightly bind to cellular proteins [70]. It is not clear how PT modification affects DNA; however, this modification makes the oligonucleotides more hydrophobic and thereby increases off-target effects with proteins and might cause toxicity [71].

Peptide nucleic acid (PNA) is another analogue that mimics DNA (Figure 4, structure **7**). Instead of a deoxyribose phosphodiester backbone, PNA has a pseudo-peptide backbone (**3** compared to **7**). It shows high stability and is resistant towards enzymatic degradation by nucleases. It has a good binding affinity with an excellent thermal stability, which allows formation of PNA/DNA and PNA/RNA duplexes. In live cell experiments, PNA needs to be injected into the cells [72].

Another approach to backbone modification is morpholino oligonucleotides (MOs). These are morpholino ring-based oligonucleotide analogues with a non-ionic phosphoramidate inter-subunit linkage (Figure 4, structure **8**) [73]. Incorporated into MBs they show negligible toxicity, good stability and high specificity for the target. Moreover, MOs have potential as new agents for in vivo clinical research. A brand new approach to backbone modification is the incorporation of pyrene into the phosphate backbone. These probes are able to induce a low background fluorescence signal by quenching of pyrene fluorescence from nearby nucleobases. Being incorporated into different positions of oligonucleotide probes, pyrene has been used for SNP discrimination. This form of incorporation showed excellent SNP properties for detection of the V600E mutation in *BRAF* [74].

Overall, modification of the backbone improves target recognition and stability of probes [45,46,67,68,69,70,71,72,73]. These modifications find multiple applications in SNP detection. However, the key to successful SNP detection is the fluorescence properties of the FRET pair [11,12,15,16,17,18]. Thus far, SNP detection by FRET has been based exclusively on measurements of intensity difference [63]. The energy transferred can be measured quantitatively so a stoichiometric ratio can be obtained. This requires photostability of both the acceptor and the donor, since bleaching in one or both fluorophores might lead to non-stoichiometric conditions [11,12,15,16,17,18]. Nowadays, many dyes suffer from a low quantum yield and brightness [2]. This can be improved using bright fluorescent dyes and materials. Among others, it can be lanthanide chelates, fluorescently labelled organic polymers such as oligo ethylene glycol methacrylate (OEGMA) nanoparticles, and QDs [2]. Noteworthy, optical properties crucially depend on the purity of the aforementioned molecules [16]. An alternative approach for increasing fluorescence response has been achieved through energy transfer between more than two fluorophores [75].

Another important parameter that might affect FRET is the attachment of fluorophores to probes. It may be typical to think that when the linker between the nucleic acid and the dye is long enough, the orientation factor is estimated to be 2/3, however, Quellet et al. [76] showed that longer linkers might adopt orientations more energetically favorable and avoid steric hindrances which may contribute to a further degree of uncertainty of the orientation of the transitions dipoles. Additionally they concluded for cyanine dyes that terminal stacking is an intrinsic property and not correlated to length of the tether. Ranjit et al. [77] showed that a rational rigid linker gave rise to a better FRET efficiency due to a locked orientation of the dyes and their transition dipoles, and the prevention of undesirable dye-DNA interactions.

Having outstanding brightness and multifunctional design, semiconductor QDs are currently a blockbuster in the toolbox for fluorescence imaging [78]. A QD typically consists of a core made of inorganic material such as Cd and Se. To improve optical properties it can be surrounded by a semiconductor shell, e.g., made of ZnS. Besides excellent brightness, QDs have broad absorption and narrow emission spectra. QDs do not suffer from photobleaching and have a high quantum yield, but sometimes suffer from blinking and photo-brightening [78]. Donor and acceptor nucleotides can be attached to the QD where it can also serve as light harvester and initial donor. A dual-color photochromic FRET system with a central QD called a concentric FRET (cFRET) has been recently reported [79]. The authors underlined though that FRET effects can involve multiple steps in such systems. Noteworthy, in order to display sufficient optical properties, QDs have to be of high purity [78]. The lifetime of the FRET donor is also able to affect the efficacy of FRET. Luminescent lanthanide complexes have shown to have a long excited-state lifetime, which can be utilized in Time-Resolved FRET [80,81]. One excellent example of this approach is the luminescent terbium complex Lumi4-TB (Tb). For microRNA detection Hildebrand and colleagues have taken advance of Tb in crosstalking multiplexes systems such as Tb-to-QD FRET complexes and Tb-to-dye hybridization/ligation based complexes [81,82].

#### 2.3.3. Up-to-Date Applications of FRET Probes: Summary and Specific Cases

Table 1 summarizes the recent application of FRET probes for nucleic acid detection, including SNP analysis. As one can see, many probes are now chemically modified at the backbone. Growing applications in fluorescence microscopy put requirements on the dyes; therefore, more photostable variants find applications [48,55,74,82,83,84,85,86,87,88]. Another important aspect is growing requirements for multiplexing in vitro and in vivo, i.e., demand for a broader spectrum of excitation and emission wavelengths provided by FRET pairs [74,81]. Based on our evaluation of the literature for 2010–2016, FRET probes take approximately 70% of all enzymatic genotyping assays (e.g., FISH, PCR), where they have confirmed superiority to all other methods. An ultimate challenge for modern FRET probes is, however, imaging in living cells [48,55].

Among other FRET pairs, the particular advantage of the pyrene-perylene donor-acceptor pair is its high quantum efficiency, permitting higher sensitivity of measurements. We applied a pyrene-perylene FRET probe for detection of HIV-1 SNP in vitro and in cell culture. However, a drawback of pyrene is the requirement of near-ultraviolet excitation light, which can damage living cells [89]. A new approach has been developed for the design of deep-red to near infrared fluoresce emitting agents with large Stokes shifts and a good photostability [90,91].

Instead of traditional MBs, new research proposes MBs containing multiple fluorophores [75]. Such probes exhibit a unique FRET, through a cascade of energy transfers [15]. As in rational MB, the fluorophores light up upon hybridization to the target. According to the design, dyes are arranged along the probe in order of increasing absorption/emission wavelength, as this allows for efficient spectral overlap and hence, FRET. This enables a maximal light outcome to be obtained and improves the detection limit. Nevertheless, false positive results provided by multi-dye MBs are still to be addressed [75].

**Figure 5:**
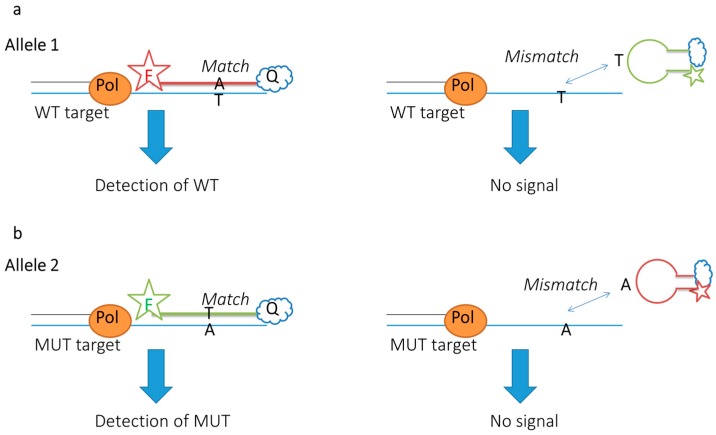
SSchematic representation of allele-specific PCR using FRET probes. WT = wild-type (**a**) and MUT = mutant (**b**) targets; Pol = polymerase. Signal is increased upon amplification of the specific allele.

The energy transfer probes used to monitor DNA amplification in PCR are either cleaved in the reaction (as with TaqMan probes), incorporated into amplified DNA (as with Scorpion primers) or undergo a conformation change in the presence of a complementary DNA target (as with MBs) [2]. As mentioned above, most SNP-sensitive designs nowadays apply the MB principle, also in a highly multiplexed fashion. In each case, the probes’ unique signal occurs upon PCR target amplification by eliminating the quenching influence on the donor fluorophore. Examples of FRET PCR are given in Table 1. It is possible to distinguish between alleles with the use of binary FRET probes [56]. This is done using a FRET system which is able to discriminate between wild type (WT) and mutant type (MUT) targets. One donor probe is enough as long as it is able to interact with both acceptors. The acceptors must differ in affinity to WT and MUT targets [56]. Allele specificity can also be obtained with MBs by using more than one probe (Figure 5a,b) [92]. One probe should be antisense to the WT while the other should be antisense to the MUT. In both designs, PCR is required to enhance the signal intensity. If the probes are designed successfully, fluorescence intensity will only increase for a perfect complementary probe match. Genotyping can be done by comparing the intensity with a known intensity of both alleles: R = FA1/(FA1 + FA2) [92].

In spite of all the aforementioned, applying FRET probes in vivo is still a very complicated affair. Based on current knowledge, the optimal oligonucleotide probes for imaging in cells might have a two-photon organic dye with a broad excitation band and be attached in the right orientation with respect to the acceptor FRET. Herein, nanoparticles such as QD are very promising [2]. However, their toxicity must be reduced, whereas uptake efficacy still needs to be improved [93]. Generally, probes which have been modified at the backbone show improved stability and specificity of target binding, but at the cost of decreased permeability [60]. This means that they need be injected. Alternatively, chemical assistance can be applied to transport probes into cells. Streptolysin-O (SLO) is an example of a reversible agent that improves cellular update of backbone modified oligonucleotides [94].

Another promising approach for probe delivery is nanoparticles [94]. The surface composition and the size of the nanoparticles loaded with probes can be modified to exactly match the required properties such as cell-specific uptake and endosomal release. Some examples include gold nanoparticles for delivering MBs, coated polymeric beads and PNA-DNA particles [88,94].

### 2.4. Computational Strategies Help Designing Efficient FRET Probes

Modern computational strategies have much to offer to the field of SNP-sensitive FRET probes. This is mainly due to the fact that a better understanding of the fluorophores on a molecular level is essential for improvement of the probes, and exactly such a detailed atomistic insight into the FRET process can be obtained by computational models. The green fluorescent protein (GFP), and related fluorescent proteins, are today among the best described fluorophores for DNA/RNA binding proteins [95,96], and can be applied as excellent FRET donors and acceptors. Through a reliable description of the proteins it has, for example, recently been possible to study key FRET mechanism parameters such as transition dipole moment orientation and the distance, r, between the acceptor and the donor [16,95]. Since FRET is based on excitation and emission processes of fluorophores from GS and EX, an appropriate description of both these states is required. Today, the most used ab initio method for modeling the GS is density functional theory (DFT). Even though DFT is not the most accurate quantum chemical model it usually predicts reliable results with a fairly low degree of computational effort, compared to more accurate method such as e.g., Couple Cluster [97]. DFT is a ground state quantum chemical method aimed at describing the electron density of the molecule in question. Generally, the calculation of excited states is more challenging than ground state calculations which is due to excited states being multiconfigurational. A convenient method that can be used to address excited states and their properties is time-dependent DFT (TD-DFT), which can be used to calculate absorption from the ground state, transition dipole moments, as well as emission parameters.

When aiming at performing DFT or TD-DFT calculations of fluorophores transition properties a basis set and an exchange-correlation functional is required. Here, the basis set is used to represent the electronic density and the exchange-correlation functional introduces effects of exchange and correlation into the calculations. Both the basis set and the exchange-correlation functional represent key computational aspects that must be chosen carefully in order to finally arrive at accurate molecular parameters entering into the expression for the Förster transfer efficiency. Furthermore, environmental effects such as a solvent or a biological environment must also be taken into account in the computations. Due to the fairly high scaling of the computational time and large system size in quantum chemical approaches, the environment is in such calculations often accounted for via classical mechanics leading to the so-called combined quantum mechanics/molecular mechanics (QM/MM) methods [98,99,100,101,102,103]. In such methods the quantum chemical description is limited to include only the fluorophores whereas the surroundings are described using molecular mechanics often in the form of a force field description such as e.g., the AMBER or OPLS force fields. By this it becomes possible to study very large systems at a fairly high level of accuracy.

**Figure 6:**
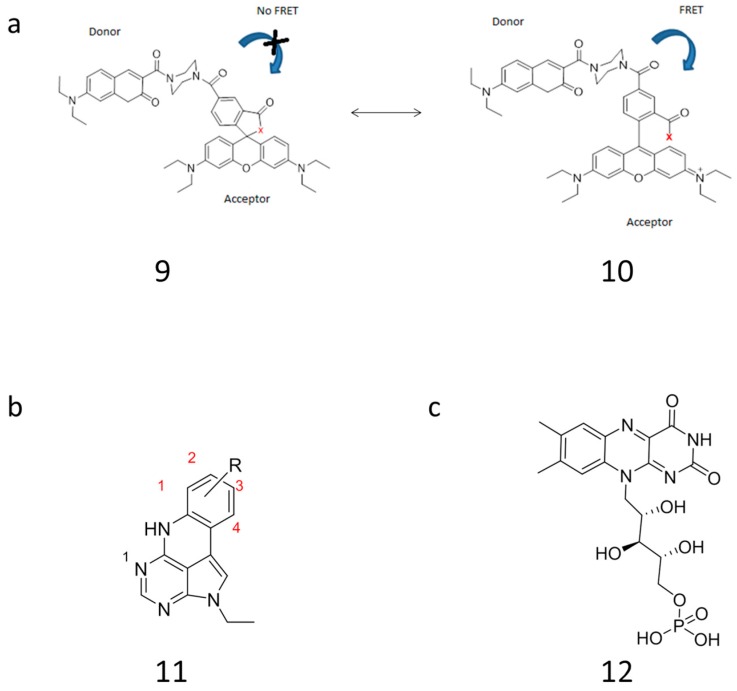
Computational design of new FRET dyes: (**a**) Rhodamine undergoes a very characteristic absorption change between the ring opening and the ring-closing conformation. Yuan et al. improve this mechanism for imagining of small molecular targets such as Cu^2+^, NO, HOCl by separation of the interaction site (denoted x) and the energy donor [103]; (**b**) Quadracyclic adenine analogues with the different substituents, R, was introduced by Larsen et al. Adenine analogues substituted at position 1 and 2 with cyanogroups showed a stable fluorescence quantum yield and environment-sensitive emission. Both properties make them suitable for monitoring nucleic acids systems [104]. R: fluorine-, methoxy- and cyanogroups; (**c**) Molecular structure of flavin mononucleotide (FMN) [105].

One successful example of optimizing FRET properties was recently described by Yuan et al. (Figure 6) [104]. In that work, a rhodamine-based probe was optimized by modulation of the spectral overlap. This was done by rationally changing the acceptor molar absorbance coefficient (Equation (4)). A rhodamine core typically contains two carboxylic acid groups. One of the carboxylic groups is able to make a ring-opened and a ring-closed system (structures **9** and **10**, Figure 6a). This interaction affects the spectral overlap and impact of the rhodamine as a FRET acceptor, due to a significant change in fluorescence for the ring-opened versus ring-closed structure. In order to avoid disruption of the rhodamine structure, Yuan et al. moved the interaction with the acceptor FRET away from the rhodamine interaction site. This resulted in optimized spectral properties and, hence, improved sensitivity of target detection [104].

Fluorescent nucleobase analogues are other possible FRET donors and acceptors [105]. Even though many currently developed emissive nucleobase analogues have a low brightness and photostability, their advantage is outstanding sensitivity to environmental changes such as SNP. To improve brightness and quantum yields of these promising molecules chemical computations can be very useful. In a recent example, Larsen et al increased the brightness of nucleobase analogues based on experimental and theoretical TD-DFT calculation of quadracyclic adenine analogues **11** (Figure 6b) [105]. The authors underlined the broad potential of the developed method to other nucleobase analogues for potential applications in FRET pairs.

List et al [106] studied the effect of one- and two-photon absorption in Flavin mononucleotide (FMN) (compound **12**, Figure 6c), a molecule structure with some similarities to xanthene derivatives such as FAM, TAMRA and fluorescein. The environmental effects on FMN was studied experimental and computational in a solvent and in a protein in order to examine the effect of non-linear two-photon absorption in a dynamic environment compared to static environment (a protein). To account for environmental effects polarizable embedding [107,108,109] was employed. The consistency between the experimental and computational results indicated a reliable use of such a computational method to describe fluorophore in complicated local environments.

## 3. Conclusions

Mutation-specific probes which use FRET have been successfully applied in the life sciences since the 1990s. Over this period, multiple probe designs have been developed. In general, single-stranded linear, hairpin-shaped or double-stranded probes are most often applied. Among these, hairpin-shaped probes represent one of the most popular designs for SNP detection in vitro and in vivo. However, multiple factors affect the performance of FRET probes in vitro and especially in vivo. These include, but are not limited to, the positioning, number and chemistry of fluorophores, as well as the incorporation of specific recognition and DNA enzyme sequences, all components used in the construction of new probes.

Chemical modification of DNA and RNA probe backbones improves specificity of SNP recognition. In particular, LNA has a profound effect on the affinity and, most importantly, specificity of target recognition.

An important aspect of FRET, making it an attractive technology in cellular research, is its ability to detect and monitor many of the reactions in live cells. It makes this approach a valuable tool for future in vivo visualization of cellular processes. This direction is rapidly developing and is putting new demands on the rational design of FRET probes. Another important application of FRET probes is the real-time monitoring of DNA amplification in PCR. Utilization of FRET permits the simultaneous detection of multiple products and is highly suitable for clinical diagnostic applications.

The use of computational strategies is a rapidly developing research area which carries great promise for mutation-sensitive FRET probes. This novel direction in probe design develops our fundamental understanding of fluorophores’ interaction with each other and with nucleic acids. Besides a deeper knowledge of nucleic acid structure, optical properties and dynamics, computational strategies lead to the successful design of successful probes in silico, i.e., prior to synthesis. The resulting probes are not restrained by the limitations inherent to existing rational design principles but instead apply fundamental knowledge of biomolecular interactions in order to gain more versatile energy transfer and, hence, excellent target detection.

## Figures and Tables

**Table 1:** Representative assays and FRET probe design for nucleic acid detection ^1^.

Probe Design & Sequence (5′-3′)	Target/Assay	FRET Dyes	Backbone ModiFication	LOD	SNP Detection	Ref.
***Unimolecular***						
**MB with 3 dyes at 5′ end**:d(GCU GAG AAG TTA GAA CCT ATG CTC AGC)	cDNA/in vitro hybridization	Terminal: Pyrene, FAM, TAMRA, Q: EB	None	1 fM	+	[74]
**In-stem MB**: d(GXTG GXTG CCA GGG CAG TGA TCT CTC CAQQC CAQQC)	*β*-actin mRNA/FISH	X = Cy3; Q = Nitro methyl red	None	0.2 μM	+	[83]
**PNA-MB**: H-Lys-(A)-GTCC GYA-Arg(TO)-ATAGCCG-Gly-NH2	cDNA/in vitro hybridization	TO, ICC	PNA	40 pM	−	[26]
**In-stem LNA-MB**: d(GGT CXX CTA GAG GGG TCA GAG GAT QQG ACC)	cDNA/in vitro hybridization	X = Pyrene, Q = PDI	None	0.3 nM	+	[84]
**MB with LNA in the loop**: d(CCGACT ATCTGCACTAGATGCACCTTAC/Bio/CGG)	Serum miRNA/qRT-PCR	Terminal: FAM, Q: 3Dab	LNA, biotin	0.5 μM	−	[85]
***Bimolecular***						
**BP with three dyes**: Cy5-r(GUA UGU UUC ACU GGA UGA), r (AAG UGG AUC AAG dT(FAM)UG GU(TAMRA)	Sensorin mRNA from neurons/in vitro hybridization	FAM, Cy5, TAMRA	2′-OMe-RNA	26 nM	−	[55]
**OP PNA-BP**: d(CTCTTCTU(FAM)TTTT CCT)-K, K(Cy5)-TCC CTC TTC CG ATC	cDNA/in vitro hybridization	Cy5, FAM	Protected PNA	0.2 μM	−	[86]
**BP—Bispyrene**: bis-Pyr-r(GAG CCG AUU UCA UCA)T, r(GGA GAA GGU GUC UGC GGA G) bis pyr	SNP C677T in MTHFR gene/in vitro hybridization	bis-Pyrene	2′-OMe-RNA, 3′-inverted thymidine	Nd	+	[87]
**PNA-DNA BP**: d(TCT TCA CGT TGT TGT)-K-(ε)-Cy5, FAM-(ε)-K-d(ATG TCC TTT TCC TCT)	iNOS mRNA/cell line study	Cy5, FAM	PNA	2 μM	−	[88]
**PAH-DNA BP**: X-CT^L^ TCC AC^L^A, CA^L^C CAA C-Y	HIV-1 RNA/cDNA/in vitro hybridization and RT-qPCR	Pyrene, Perylene	2′-amino-LNA, LNA	5 nM	+	[48]
**Tb/fluorophore miRNA-complex**: Tb-CGA TCA GTC-AGG-CAA-AGC-GG, TTA-CTG-TGC-ACA-GAG-GA-X	Colon-adeno-carcinoma-Hsa-miR-20a-5p, in vivo hybrdization and ligation	Tb; X = Cy3.5	5′ C6 thiol, 3′ C7 amine	0.2 nM	+	[82]

^1^ nd = no data; MB = molecular beacon; BP = binary probe; PNA = peptide nucleic acid; LNA = locked nucleic acid; TO = thiazole orange; PAH = polyaromatic hydrocarbon; Pyr = pyrene; PDI = perylenediimide; LOD = limit of target detection; OP = orthogonally protected; Tb = terbium complex Lumi4-Tb.
